# Predicting violence in female forensic inpatients with substance use disorders – the utility of a gender-responsive assessment

**DOI:** 10.3389/fpsyt.2024.1346815

**Published:** 2024-01-29

**Authors:** Viviane Wolf, Juliane Mayer, Ivonne Steiner, Irina Franke, Verena Klein, Judith Streb, Manuela Dudeck

**Affiliations:** ^1^ Department of Forensic Psychiatry and Psychotherapy, kbo-Isar-Amper-Clinic Taufkirchen (Vils), Taufkirchen (Vils), Germany; ^2^ Department of Forensic Psychiatry, Psychiatric Services of Grisons, Chur, Switzerland; ^3^ Department of Forensic Psychiatry and Psychotherapy, Ulm University, Guenzburg, Germany

**Keywords:** risk assessment, violence, recidivism, justice-involved women, substance use disorder

## Abstract

**Introduction:**

Given that risk assessment tools are commonly based on male samples, the applicability to justice-involved women remains to be clarified. This study aimed at assessing (1) the predictive validity of the HCR-20 V3, the prevailing, yet primarily male-based violence risk assessment instrument, and (2) the incremental validity of the FAM, a gender-responsive supplement, for both inpatient violence and violent recidivism in justice-involved women.

**Methods:**

The sample included 452 female forensic inpatients with substance use disorder discharged from German forensic psychiatric care between 2001 and 2018.

**Results:**

ROC analyses revealed good predictive accuracy for the HCR-20 V3 while the FAM failed to provide incremental validity. Further, binary logistic regression determined several predictors of violence including personality disorder, covert/manipulative behavior, suicidal behavior/self-harm, and problematic intimate relationship.

**Discussion:**

These findings support the applicability of the HCR-20 V3 in justice-involved women with substance use disorder, while highlighting the clinical relevance of the FAM in supporting a gender-informed risk management.

## 1 Introduction

Despite being subject to extensive research throughout the past few decades, the literature on violent offending is heavily skewed toward the male gender. As with other forms of antisocial behavior, being male is considered a central predictor of violent behavior. Consequently, a considerable gender gap emerged, with empirical studies largely failing to address female violence. However, though merely accounting for a small percentage (6% to 10%) of prison and forensic psychiatric populations, globally a notable increase in violent crimes and admission rates to forensic psychiatric care has been recorded for women in the last decades (1–5). The latter trend can partially be attributed to the higher prevalence of mental disorders among justice-involved women, resulting in more diminished criminal responsibility outcomes compared to men (1, 4, 6). The relevance of gender in criminality research is further underscored by recent studies highlighting several gender differences in the nature of offending and the associated risk factors. Primarily, various forms of violence were found particularly prominent among justice-involved women, including inpatient violence, child abuse, and intimate partner violence (3, 7). Further, women tend to use violence in a more reactive and indirect manner compared to men, often within social relationships and for less of an instrumental purpose. Also, women frequently claim motives such as jealousy, self-defence, and feelings of disrespect and are more inclined than men to use personal weapons (e.g., hands, teeth, or knives) when engaging in violent behavior (8).

Similarly, a growing body of feminist literature emphasizes the unique pathways to female criminality, suggested to be shaped by gendered and disadvantaged life circumstances (9–12). Accordingly, researchers have identified several gender-responsive risk factors of female (re-)offending including mental health issues (9, 13, 14), trauma/abuse (9, 13, 15), low self-esteem/self-efficacy (9), parental stress (9, 14, 16), and intimate partner dysfunction (9, 13, 17, 18). Additionally, factors such as addiction and poverty have been found to be more pronounced in women (13, 14, 19, 20). With regard to female violent offending, Mackey (21) concluded intimate partner violence to be associated with child abuse, substance/alcohol use, borderline personality traits, attachment issues, and trauma. Similarly, Warren (22) found Cluster B personality disorder to be related to violence in women. Herrera (23) determined that women who had endured physical abuse during their childhood were seven times more prone to engage in violent offenses compared to their non-abused counterparts. Another study examining gender differences in violent offending also revealed lower educational achievement, adverse childhood experiences, and mental health issues to be more prevalent in women (24). Further risk factors include self-harm (25), low self-esteem (8) and alcohol use disorder (26).

Evaluating the risk of future violence is a crucial component of forensic psychiatric treatment (27). To standardize the process and improve accuracy, violence risk assessment instruments are commonly employed (28). The Historical Clinical Risk Management-20, Version 3 (HCR-20 V3) (29) is currently the prevailing violence risk assessment tool, comprising both static (i.e., fixed) and dynamic (i.e., changeable) risk factors of future violence. Extensive research confirms robust predictive accuracy in diverse populations (30). However, studies on its effectiveness with justice-involved women have yielded inconsistent findings (31–36). A meta-analysis by Rossdale (4) concluded low to moderate effect sizes, while demonstrating that only two of the 12 investigated studies incorporated the third version of the HCR-20. Further, like other current risk assessment tools, the HCR-20 V3 faced criticism for relying on risk factors derived from predominantly male samples (1), thereby failing to recognize risk factors sensitive to females (9, 12, 37). To address this issue, the Female Additional Manual (FAM) (8) was designed as a gender-responsive supplement to the HCR-20. While primary studies could only demonstrate the clinical relevance of the FAM (27, 35), at present insufficient research is available to permit conclusive statements on its predictive accuracy (38). Further, existing studies are lacking specificity concerning the variety of mental disorders. In fact, research has highlighted that violence risk strongly varies across psychiatric diagnoses. Being among the most violence-prone mental disorders, substance use disorders (SUD) are in particular need of empirical evidence on the applicability of risk assessment tools (39). Equally, the HCR-20 V3 has yet to be validated for this subgroup of justice-involved women.

Incorporating a retrospective follow-up design, the given study aimed to substantiate the applicability of the HCR-20 V3 in female forensic inpatients with SUD. Equally, it aimed to evaluate the incremental validity of the FAM as a supplementary gender-responsive assessment. For these purposes, the predictive accuracy of both tools was determined, in separate and combined application. Additionally, the instruments’ individual items were analyzed to determine risk factors for female violence. Two violent outcomes (i.e., inpatient violence, violent recidivism) served as dependent variables.

## 2 Materials and Methods

### 2.1 Participants

The given study included 452 female forensic psychiatric inpatients discharged from forensic psychiatric care in Bavaria, Germany, between 2001 and 2018. To be eligible for inclusion in the study, patients had to be at least 18 years of age at discharge and have a final conviction causal to admission to a forensic psychiatric facility. All patients were court-ordered to undergo forensic psychiatric treatment, either according to section 63 or section 64 of the German Penal Code. To be admitted according to section 63, a severe mental disorder is assumed to be decisive of the initial offense. Further, a considerable risk of reoffending and diminished criminal responsibility is presumed. The duration of treatment is not time-limited but is annually reevaluated by a forensic psychiatric professional. To be admitted according to section 64, a substance use disorder is considered to have centrally contributed to the initial offense. Admission further requires a considerable risk of reoffending and a positive treatment prognosis. The length of treatment is limited in time and generally amounts to two years but may vary depending on additional custodial sentences. If the criteria for successful treatment are no longer met, patients admitted according to section 64 may return to prison. In this context, successful treatment concerns the achievement of individual therapeutic aims that serve the overarching goal of minimizing the patients’ recidivism risk while facilitating their successful reintegration into society. Commonly, such objectives entail abstinence from substances, alleviation of psychiatric symptoms, enhanced social functioning, insight into mental disorder and gradual reduction of the levels of restriction, eventually leading to dismissal from forensic psychiatric treatment (40).

### 2.2 Materials

#### 2.2.1 Historical clinical risk management-20, version 3 (HCR-20 V3)

As a standardized violence risk assessment tool that incorporates a Structured Professional Judgement (SPJ) approach, the HCR-20 V3 is designed to assist in the prediction and prevention of future violence. Additionally, it provides appropriate risk management strategies. The tool comprises 20 research-based risk factors, organized into three domains. The historical domain (H) contains 10 items related to past problems (i.e., violence, antisocial behavior, relationships, employment, substance use, major mental disorder, personality disorder, traumatic experiences, violent attitudes, and treatment/supervision response). The clinical domain (C) includes five items that pertain to problems in the last six months, including insight, violent ideation/intent, symptoms of a major mental disorder, instability, and treatment/supervision response. Finally, the risk management domain (R) includes five items that concern anticipated problems in the next six months, including professional services/plans, living situation, personal support, treatment/supervision response, and stress/coping. All risk factors are coded on a 3-point scale (present/partially present/not present). Additionally, the individual relevance of each risk factor to the assessed patient is evaluated to allow for more personalized risk assessment and treatment planning. A review by Douglas (30) reported acceptable interrater reliability for the HCR-20 V3, while more recent findings even determined good to excellent interrater reliability (41).

#### 2.2.2 Female additional manual (FAM)

As a supplement to the HCR-20 V3, the FAM is a gender-responsive risk assessment tool that provides additional risk factors and guidelines tailored to justice-involved women. Notably, the FAM was originally designed to supplement the second version of the HCR-20 and subsequently modified to be used alongside the third version of the HCR-20. It comprises two supplementary guidelines and eight additional risk factors, which are divided into four historical factors (i.e., parenting difficulties, suicidality/self-harm, prostitution, and pregnancy at a young age), two clinical factors (i.e., covert/manipulative behavior and low self-esteem), and two risk management factors (i.e., problematic childcare responsibility and problematic intimate relationship). For the individual items and additional guidelines, good interrater reliability was found (ICC = .63-.97) (8).

### 2.3 Procedure

The given study was part of an extensive research project evaluating the applicability of common risk assessment tools in female forensic inpatients. In this study, we aimed to predict violence in female forensic inpatients with SUD by applying two violence risk assessment instruments. First, the HCR-20 V3 was used to account for general risk factors of violence. Second, the FAM was incorporated to add gender-responsive risk factors of violence. Coding was performed by five researchers, who underwent professional training in preparation for the application of the instruments. Subsequently, interrater reliability testing was conducted to assess the interrater agreement on the included violence risk assessment instruments. The HCR-20 V3 yielded moderate results (ICC = .606, 95%-CI = .345;.845), while good results were obtained for the FAM (ICC = .818, 95%-CI = .638;.938). The items were rated using file information retrieved from patient records, including official court documents. As files differed in quality and completeness, only those patient files were included in the study that provided the necessary information to properly assess the items (i.e., at least the court decision/forensic psychiatric assessment at admission and a report on the therapeutic process at discharge). The dependent variables (i.e., inpatient violence, violent recidivism) were coded on a binary scale (yes/no). Any interpersonal violence during forensic treatment was coded as inpatient violence and any violent reconviction was considered violent recidivism. Violence was assessed in accordance with the HCR-20 V3 definition, which is the “actual, attempted, or threatened infliction of bodily harm on another person”. To assess violent reoffending, extracts from the Federal Central Criminal Register were obtained in September 2020 and February 2021. Data collection took place between 2019 and 2021 and the study was approved by the Ethics Committee of the Bavarian Medical Association (approval no. 2019-167).

### 2.4 Data analysis

All statistical analyses were performed using IBM SPSS Statistics Version 29. Missing data was minimal (range: 0% to 1%) and randomly distributed (MCAR), so no imputation methods were employed, and participants with missing values on any examined items were excluded from the analysis. First, binary logistic regression was conducted to determine significant (*p* <.05) predictors of both violent outcomes (i.e., inpatient violence, violent recidivism) among the individual items of the risk assessment instruments. Next, receiver operating characteristic (ROC) analysis was conducted to determine the predictive accuracy of the HCR-20 V3 (subscales and total score) and the FAM (total score) for the violent outcomes. For the interpretation of AUC values, the guidelines provided by Rice and Harris (42) were applied. Accordingly, values greater than .56 were classified as small effects, those above .64 were considered medium effects, and values exceeding .71 were deemed large effects.

## 3 Results

### 3.1 Sample characteristics

For the total study sample (*N* = 452), the mean age at admission was 33.16 years (range: 17-61 years), while the mean duration of inpatient treatment was 27.32 months (range: 0-152 months) and the mean follow-up period was 8.84 years (range: 2.05-19.05 years). Most of the patients (*N* = 415, 91.8%) were admitted according to section 64 of the German Penal Code, with the remaining 8.2% (*N* = 37) being admitted according to section 63. Regarding the psychiatric diagnoses at the time of discharge, 26.3% were diagnosed with an alcohol use disorder (ICD-10, F10) and 83.6% with other substance use disorders (ICD-10, F11 – F15). Concerning comorbid diagnoses, 4.4% were diagnosed with an affective disorder (ICD-10, F3), 3.5% with a neurotic disorder (ICD-10, F4), and 3.8% with an eating disorder (ICD-10, F5). Further, 21.2% were given the diagnosis of a personality disorder (ICD-10, F6), with emotionally unstable personality disorder being the most common subtype (17.9%), followed by mixed (4%) and antisocial personality disorder (2%). Concerning the initial offenses, 36.9% of the patients committed a violent offense. Among the violent offenders, 5.3% were convicted of homicide, 19.9% of bodily harm, 4.2% of arson, 6% of robbery, and 1.1% of unlawful detention/threat/coercion. Among the non-violent offenders, 44.5% were convicted of drug-related offenses, 14.6% of property offenses, and 4.4% of other, non-violent offenses. Concerning inpatient violence, 8% (*N* = 36) of the sample engaged in interpersonal violence during detention. Regarding recidivism after discharge from forensic psychiatric care, 43.6% (*N* = 197) reoffended, while 12.2% (*N* = 55) reoffended violently.

### 3.2 Predictive utility of the individual items on violent outcomes

#### 3.2.1 HCR-20 V3

As displayed in **Table 1**, binary logistic regression analyses determined several significant predictors for both violent outcomes including the historical factors violence, personality disorder, violent attitudes and problems with treatment/supervision. Regarding the clinical and risk management factors, each factor, except for recent symptoms of a major mental disorder, was found a significant predictor for violent recidivism. In contrast, inpatient violence was significantly predicted by all clinical and risk management factors, except for future problems with professional services and personal support.

**Table 1:** Binary logistic regression analyses predicting violent outcomes based on individual HCR-20 V3 items.

History of/recent/future problems with…	Inpatient violence		Violent recidivism	
	Exb (B)	p	Exb (B)	p
H1 Violence	3.296	<.001	2.145	<.001
H2 Other antisocial behavior	2.966	.248	2.403	.188
H3 Relationships	1.457	.566	1.502	.454
H4 Employment	1.483	.287	2.509	.025
H5 Substance use	–	–	–	–
H6 Major mental disorder	1.402	.074	1.001	.995
H7 Personality disorder	2.700	<.001	1.738	<.001
H8 Traumatic experiences	2.044	.167	1.947	.100
H9 Violent attitudes	3.461	<.001	2.818	<.001
H10 Treatment or supervision	2.045	.046	2.933	.003
C1 Insight	1.502	.040	2.023	<.001
C2 Violent ideation or intent	4.949	<.001	2.269	.018
C3 Symptoms of major mental disorder	1.800	.004	1.319	.130
C4 Instability	2.173	.002	2.293	<.001
C5 Treatment or supervision	1.762	.006	1.852	<.001
R1 Professional services	1.430	.088	2.242	<.001
R2 Living situation	2.257	.014	2.287	.002
R3 Personal support	1.387	.178	2.327	<.001
R4 Treatment or supervision	1.925	.007	2.523	<.001
R5 Stress or coping	3.745	.003	2.690	.001

#### 3.2.2 FAM

As shown in **Table 2**, binary logistic regression analyses revealed several significant risk factors for both violent outcomes including personality disorder and covert/manipulative behavior. Further, inpatient violence was significantly predicted by suicidal behavior/self-harm, while violent recidivism was significantly predicted by problematic intimate relationship. A separate examination of the subitems of the item personality disorder showed that for both violent outcomes the subitems antisocial and other Cluster B were significant predictors, while the item other personality disorder was not.

**Table 2:** Binary logistic regression analyses predicting violent outcomes based on the individual FAM items.

	Inpatient violence		Violent recidivism	
	Exp (B)	p	Exp (B)	p
H7 Personality disorder	4.119	<.001	2.401	<.001
H8 Traumatic experiences	2.040	.168	1.943	.101
H11 Prostitution	1.348	.170	.680	.143
H12 Parenting difficulties	.742	.119	.868	.347
H13 Pregnancy at young age	1.008	.964	1.122	.440
H14 Suicidal behavior/self-harm	2.550	<.001	1.337	.059
C6 Covert/manipulative behavior	1.506	.042	1.637	.004
C7 Low self-esteem	1.198	.398	1.159	.399
R6 Problematic child care responsibility	.874	.507	1.014	.931
R7 Problematic intimate relationship	1.103	.683	2.085	.003

### 3.3 Predictive validity of HCR-20 V3 and FAM on violent outcomes

#### 3.3.1 Inpatient violence

The results from Receiver Operating Characteristic (ROC) analysis, as shown in **Table 3**, revealed that all measures significantly predicted inpatient violence. However, large effects (AUC >.71) were found only for the historical scale and the total score of the HCR-20 V3, both with and without addition of the FAM. Moderate effects (AUC >.64) were determined for the clinical scale of the HCR-20 V3, with and without the FAM and the risk management scale of the HCR-20 V3. For the remaining measures, only small effects were found. Regarding the supplementary assessment of the FAM, the results show a decrease in predictive accuracy. When administering the FAM without the intended additional application of the HCR-20 V3, the AUC further declined, reflecting a small effect.

**Table 3:** AUC analysis assessing the predictive accuracy of HCR-20 V3 and FAM on inpatient violence (N = 452).

	AUC	CI 95%	Standard error	p
H-Scale HCR-20 V3	.814	(.742,.885)	.037	<.001
C-Scale HCR-20 V3	.684	(.593,.775)	.046	<.001
R-Scale HCR-20 V3	.653	(.572,.734)	.041	.002
Total score HCR-20 V3	.762	(.684,.839)	.040	<.001
Total score FAM	.635	(.545,.724)	.046	.007
H-Scale FAM + HCR-20 V3	.756	(.679,.833)	.039	<.001
C-Scale FAM + HCR-20 V3	.687	(.596,.778)	.047	<.001
R-Scale FAM + HCR-20 V3	.620	(.534,.706)	.044	.017
Total score FAM +HCR-20 V3	.730	(.650,.809)	.040	<.001

#### 3.3.2 Violent recidivism

The results from the ROC analyses, as displayed in **Table 4**, demonstrate that all measures significantly predicted violent recidivism. However, large effects (AUC >.71) were found only for the historical scale of the HCR-20 V3 as well as the risk management scales and the total scores of the HCR-20 V3, with and without addition of the FAM. For the remaining measures, small to medium effects were determined. When applying the HCR-20 V3 in conjunction with the FAM, the prediction of violent recidivism remained statistically significant. However, the AUC value slightly declined, indicating that the addition of the FAM did not improve the predictive accuracy. Further, applying only the FAM without the intended assessment of the HCR-20 V3 resulted in a further decrease in the AUC value, reflecting a small effect.

**Table 4:** AUC analysis assessing the predictive accuracy of HCR-20 V3 and FAM on violent recidivism (N = 452).

	AUC	CI 95%	Standard error	p
H-Scale HCR-20 V3	.742	(.673,.811)	.035	<.001
C-Scale HCR-20 V3	.697	(.627,.767)	.036	<.001
R-Scale HCR-20 V3	.731	(.665,.798)	.034	<.001
Total score HCR-20 V3	.768	(.703,.832)	.033	<.001
Total score FAM	.614	(.539,.688)	.038	.006
H-Scale FAM + HCR-20 V3	.683	(.611,.754)	.037	<.001
C-Scale FAM + HCR-20 V3	.695	(.623,.767)	.037	<.001
R-Scale FAM + HCR-20 V3	.715	(.648,.781)	.034	<.001
Total score FAM + HCR-20 V3	.734	(.668,.800)	.034	<.001

## 4 Discussion

The present study was conducted to substitute the available knowledge on violence risk assessment in justice-involved women, particularly focusing on the population of female forensic inpatients diagnosed with substance use disorders. Our findings contribute to the existing literature by providing evidence on the applicability of the HCR-20 V3, the prevailing, yet predominantly male-based violence risk assessment tool, and the incremental validity of the FAM, the gender-responsive supplement to the HCR-20. Primarily, the recidivism rates found in the given study slightly exceed those reported in previous literature involving female forensic inpatients. While general recidivism was observed in 43.6% of the present sample, comparable studies have reported reoffending rates between 30.2% and 33.8% in mixed diagnostic samples (15, 27) and 37% in a sample exclusively considering patients with SUD (43). However, the literature on this specific population is still insufficient and has several limitations including small sample sizes and short follow-up periods. Equally, most previous studies examined samples with different psychiatric diagnoses whereas the present study exclusively focused on patients with SUD. This distinction is particularly pertinent, given that substance abuse itself constitutes a major risk factor for general recidivism (39). This conclusion is also supported by a study on female forensic inpatients with schizophrenia conducted at the same forensic facility as the present study, which reported significantly lower rates of general recidivism (i.e., 21.2%) (44).

Further, our findings validate the feasibility of the HCR-20 V3 in justice-involved women. Particularly, large effect sizes were found for the prediction of both violent outcomes (i.e., inpatient violence, violent recidivism) when applying the HCR-20 V3 (total score) to the studied population. Given these results correspond with findings in male samples (30), the HCR-20 V3 appears to perform comparably for justice-involved women with SUD and mentally disordered offenders in general. Also, it coincides with recent findings on justice-involved women accentuating the enhanced effectiveness of HCR-20 Version 3 over Version 2 in evaluating recidivism among females (27). Likewise, it supports prior studies (35, 45) using the HCR-20 V2 to predict inpatient violence in female forensic patients. Concerning the binary logistic regression results of the HCR-20 V3 items, most significant predictors were found for the clinical and risk management scales while for the historical scale only previous violence, violent attitudes, personality disorder and past problems with supervision/treatment were found significant risk factors. In contrast, the AUC results of the subscales showed that the historical scale outperformed the clinical and risk scales for both violent outcomes and even surpassed the predictive accuracy of the HCR-20 V3 total score for inpatient violence. This suggests that a limited number of historical risk factors explained a substantial part of the variance in the violent outcomes, which stresses the relevance of static risk factors in justice-involved women with SUD. Further, given the specifics of the studied population, another finding might be important to reflect on. Namely “current symptoms of a major mental illness” turned out to be the only clinical factor that did not predict violent recidivism. This corresponds with prior literature suggesting that mental illness represents a responsivity factor rather than a risk factor. While not independently predicting future violence it manifests through related risk factors (46). This seems to be particularly true for the present population of patients with SUD as substance abuse frequently serves to cope with symptoms of other mental health issues (47). Hence, our findings should not be construed as diminishing the relevance of this clinical factor. Regarding the feasibility of a gender-responsive assessment, as operationalized by the FAM, mixed results were gathered for the given population. Primarily, the total score of the combined assessment of FAM and HCR-20 V3 significantly predicted both violent outcomes with large effect sizes. However, incorporating the FAM items into the HCR-20 V3 did not improve its predictive accuracy for any violent outcome, even resulting in a decreased AUC value. This finding aligns with prior studies on inpatient violence (35, 45). Also, it supports earlier findings emphasizing the reduced effectiveness of the FAM when applied in conjunction with the most recent version of the HCR-20, given its original purpose was to supplement the second version of the HCR-20 (27). However, certain FAM items significantly predicted violent outcomes but differed depending on the measure of violence. While both measures of violence were predicted by personality disorder (i.e., antisocial, other Cluster B) and covert/manipulative behavior, suicidal behavior/self-harm also predicted inpatient violence and problematic intimate relationship was determined a risk factor for violent recidivism. These findings correspond with prior research on female criminal (re-)offending that found intimate relationship dysfunction, self-harm and personality disorders (i.e., mixed, antisocial) to be significant risk factors (9, 14, 25). Equally previous literature on female violent (re-)offending found significant associations for borderline personality traits and Cluster B personality disorder (21, 22). Lastly, the demonstrated link between inpatient violence and self-harm aligns with recent findings highlighting the complex risk profile that goes along with the co-occurrence of self-harming behaviors and violence, also referred to as “dual harm” (48). Equally, it corresponds with recent findings on a Dutch mixed-gender sample that found self-harm to be the central predictor of inpatient violence in forensic psychiatric patients (49).

In sum, it can be recognized that the HCR-20, with and without supplementary assessment of the FAM, showed higher predictive accuracy in the given population when compared to most of the previous studies on female forensic inpatients (4, 27, 32). This difference may be explained by several factors. First, among the limited available studies on this topic, very few studies have assessed the latest version of the HCR-20, which was applied in the present research. Equally, existing studies have either used relatively small sample sizes and/or short follow-up periods (4). Lastly, the diagnostic focus on SUD chosen by the present study may have impacted the results. In fact, a similar study, conducted at the same forensic clinic during the same treatment period, assessed the predictive accuracy of the HCR-20 V3 and the FAM in 99 female forensic inpatients diagnosed with schizophrenia spectrum disorders and produced considerably different results (44). Particularly, the prediction of violent recidivism using the HCR-20 V3 produced only moderate effect sizes and the additional assessment of the FAM did not reach significance at all. Equally, no significant associations between the FAM items and violent outcomes (i.e., violent index offense, inpatient violence, violent recidivism) were found. This indicates that the HCR-20 V3 total score and the FAM, both on item level and as a total score, perform differently depending on the patients’ psychiatric diagnoses. Particularly, its performance appears to be superior in patients with SUD than in schizophrenia patients. However, given the present study also included a higher sample size, this hypothesis needs to be verified by further research.

In terms of the clinical implications of this study, the findings at hand endorse the utilization of the HCR-20 V3 among female forensic inpatients with SUD. However, caution is advised in generalizing findings to different psychiatric populations, stressing the need to consider the diagnostic context when applying risk assessment tools. Further, the substantial predictive value demonstrated by the historical risk factors of the HCR-20 V3 could prove advantageous, specifically in situations characterized by limited assessment time or scarce information. Despite the FAM not improving the predictive accuracy of the HCR-20 V3, it may still hold clinical relevance in supporting gender-informed risk management and treatment planning. Particularly, it could enable a comprehensive understanding of women’s’ overall risk profiles, paving the way for more personalized risk management strategies. Especially given the instrument’s brief application period, attending to gender-responsive needs may still be a valuable addition to forensic psychiatric care (27). Conclusively, the study highlights the necessity for further research to explore variations in the performance of risk assessment tools considering different psychiatric diagnoses. This underscores the importance of a nuanced understanding of these tools in different clinical populations.

Several limitations should be considered. Firstly, we relied on retrospective record data, which was not collected specifically for this study but was obtained from existing forensic treatment documentation. Consequently, patient records varied in comprehensiveness and content. Second, due to reliance on external assessments, we were not able to gather the patient’s subjective perspective. To overcome this limitation, future research may be advised to use a mixed-methods approach. Further, the assessment of violent recidivism was limited to official offense records and more dependable inferences would have been possible if self-report data or alternative sources of information were considered. Notwithstanding these limitations, this study makes a significant contribution to the literature on violence risk assessment for justice-involved women by providing a substantial sample size, comprising all female patients with SUD who underwent forensic psychiatric treatment in one forensic psychiatric facility over a 17-year period.

## 5 Conclusion

This study presents relevant findings regarding the prediction of violence in female forensic inpatients with SUD. Primarily, the results confirm the suitability of the HCR-20 V3 for this population, revealing a good predictive accuracy for both inpatient violence and violent recidivism. Equally, large effects were found for the HCR-20 V3 in conjunction with the FAM for the prediction both violent outcomes. Notably, however, the inclusion of the FAM did not improve the predictive accuracy. Additionally, several gender-responsive risk factors of the FAM were found significant predictors of the violent outcomes. While personality disorder and covert/manipulative behavior significantly predicted both forms of violence, suicidal behavior/self-harm also predicted inpatient violence and problematic intimate relationship was found a risk factor for violent recidivism. Hence, this study substantiates the applicability of the HCR-20 V3 to justice-involved women while highlighting the clinical value of the FAM.

## Data availability statement

The raw data supporting the conclusions of this article will be made available by the authors, without undue reservation.

## Ethics statement

The studies involving humans were approved by Ethics Committee of the Bavarian Medical Association (approval no. 2019-167). The studies were conducted in accordance with the local legislation and institutional requirements. Written informed consent for participation was not required from the participants or the participants’ legal guardians/next of kin in accordance with the national legislation and institutional requirements.

## Author contributions

VW: Data curation, Formal analysis, Investigation, Methodology, Writing – original draft, Writing – review & editing. JM: Data curation, Investigation, Writing – review & editing. IS: Data curation, Investigation, Writing – review & editing. IF: Conceptualization, Writing – review & editing. VK: Conceptualization, Funding acquisition, Writing – review & editing. JS: Methodology, Writing – review & editing. MD: Conceptualization, Funding acquisition, Writing – review & editing.
